# The impact of maternal supplementation during pregnancy and the first 6 months postpartum on the growth status of the next child born after the intervention period: Follow‐up results from Bangladesh and Ghana

**DOI:** 10.1111/mcn.12927

**Published:** 2020-02-05

**Authors:** Katherine P. Adams, Seth Adu‐Afarwuah, Malay K. Mridha, Brietta M. Oaks, Susana L. Matias, Charles D. Arnold, Sika M. Kumordzie, Harriet Okronipa, Maku E. Ocansey, Kathryn G. Dewey

**Affiliations:** ^1^ Department of Nutrition University of California Davis Davis California USA; ^2^ Department of Nutrition and Food Science University of Ghana Accra Ghana; ^3^ School of Public Health BRAC University Dhaka Bangladesh; ^4^ Department of Nutrition and Food Sciences University of Rhode Island Kingston Rhode Island USA; ^5^ Department of Nutritional Sciences and Toxicology University of California Berkeley Berkeley, California USA

**Keywords:** Bangladesh, child growth, Ghana, preconception nutrition, nutrient supplements

## Abstract

Pregnancy and breastfeeding make demands on maternal nutrient stores. The extent of depletion and the degree to which nutrient stores are replenished between pregnancies has implications for a mother's nutritional status at conception of the subsequent child and therefore that child's birth outcomes and growth. Using follow‐up data collected several years after a randomized effectiveness trial conducted in rural Bangladesh and a randomized efficacy trial conducted in semiurban Ghana, we evaluated the impact of maternal supplementation with small‐quantity lipid‐based nutrient supplements (LNS) or multiple micronutrients (MMN) through pregnancy (the index pregnancy) and 6 months postpartum on the growth status of the next living younger sibling conceived and born after the index pregnancy. In both Bangladesh (*n* = 472 younger siblings) and Ghana (*n* = 327 younger siblings), there were no overall differences in the growth status or the prevalence of undernutrition among younger siblings whose mothers had received LNS (or MMN, Ghana only) during and after the index pregnancy compared with the younger siblings of mothers who had received iron plus folic acid (IFA) during the index pregnancy (Ghana) or during and for 3 months after the index pregnancy (Bangladesh). These findings do not indicate that preconception nutrition interventions do not improve child growth. Rather, they suggest that any benefits of maternal LNS or MMN supplementation during one pregnancy and for 6 months postpartum are unlikely to extend to the growth of her next child beyond any effects due to IFA alone.

**Key messages**

- Although maternal supplementation with small‐quantity lipid‐based nutrient supplements or multiple micronutrients during pregnancy and for 6 months postpartum is likely to improve maternal nutrition prior to the next pregnancy, we did not find a significant impact on the growth status of the subsequent child, compared with maternal supplementation with iron plus folic acid.
- Maternal supplementation with small‐quantity lipid‐based nutrient supplements ormultiple micronutrients during pregnancy and for six months postpartum is likely toimprove maternal nutrition prior to the next pregnancy.
- We did not find a significant impact on the growth status of the subsequent child,compared to maternal supplementation with iron plus folic acid.
- In these studies, the average gap between the end of maternal supplementation andconception of the next child was ∼15 months in Bangladesh and ∼25 months inGhana; in such scenarios, improvements in maternal nutrition may not be sustainedthrough conception of the subsequent child.

## 1 INTRODUCTION

The first 1,000 days, spanning the period from conception through 24 months of age, is often referred to as the “critical window of opportunity” for improving maternal and child nutrition (de Onis & Branca, 2016; Victora et al., 2010). Poor maternal and child nutrition during this period increases morbidity and mortality and can have adverse long‐term effects on health, cognitive development, human capital acquisition, and economic productivity in adulthood (Black et al., 2013; World Bank, 2006). For this reason, many nutrition interventions focus on improving maternal nutrition during pregnancy and/or child nutrition during the first 2 years. More recently, the preconception period has received increasing attention as another critical, and generally overlooked, window for improving maternal nutrition and child outcomes (Barker et al., 2018; De‐Regil, Harding, & Roche, 2016; Stephenson et al., 2018).

The preconception period, which is any interval of time in which a woman of reproductive age (15–49 years) is not pregnant, includes the period before first pregnancy and any interpregnancy intervals (De‐Regil et al., 2016). Several studies have shown a significant association between preconception maternal nutritional status and birth outcomes (Ramakrishnan et al., 2012; Young et al., 2015) or preconception maternal nutrition and child growth (Young et al., 2018). However, the global nutrition community has focused mainly on the first 1,000 days as the critical window of opportunity for improving birth outcomes and child growth, and the potential role of maternal nutrition during the preconception period has only recently gained attention (Bhutta, 2019). Thus, there is limited evidence on the impact of maternal nutrition interventions during the preconception period, including the importance of the specific timing and duration of the preconception intervention, on birth outcomes and child growth. A few studies have evaluated the effect of preconception nutrition interventions on birth outcomes (Hambidge et al., 2019; Nguyen et al., 2017; Potdar et al., 2014; Ramakrishnan et al., 2016). In the most recent trial, conducted in four low‐ and middle‐income countries, maternal supplementation with a lipid‐based nutrient supplement (LNS) starting at least 3 months before conception improved foetal growth, but the impact on foetal growth did not significantly differ from the impact observed when the supplement was started late in the first trimester (Hambidge et al., 2019). Whether preconception nutrition has an effect on growth measured later in childhood remains relatively unknown. One study in Vietnam showed improved linear growth at 2 years of age among children whose mothers had received preconception multiple micronutrients (MMN) or iron and folic acid compared with those who had received preconception folic acid (Nguyen et al., 2017), but more evidence is needed.

In this study, we contribute new evidence on the impact of maternal nutrient supplementation prior to conception on child growth status. Although most preconception maternal nutrition intervention trials have provided mothers with supplementation during the preconception period immediately before conception and through delivery, we explored the relationship from a different time point during the maternal preconception period. Specifically, we assessed the impact of maternal supplementation during pregnancy (the index pregnancy) and the first 6 months postpartum on the growth status of her next living child conceived after the index pregnancy. Our analyses were based on follow‐up data that were collected several years after two randomized controlled trials: the Rang‐Din Nutrition Study (RDNS) effectiveness trial in Bangladesh and the International Lipid‐based Nutrient Supplements‐DYAD‐G efficacy trial in Ghana. These trials were designed to assess the impact of combined maternal and child supplementation throughout much of the first 1,000 days on child birth and growth outcomes (Adu‐Afarwuah et al., 2016; Adu‐Afarwuah et al., 2015; Dewey et al., 2017; Mridha et al., 2016). During pregnancy and for 6 months postpartum, women who participated in the trials were provided with daily small‐quantity (20 g/day; 118 kcal/day) LNS (both trials) or MMN capsules (DYAD‐G trial only), whereas other women received daily iron‐folic acid (IFA) capsules during pregnancy and for 3 months postpartum (RDNS trial) or during pregnancy only (DYAD‐G trial). The infants born to women during the trials, referred to here as index children, also participated in the trials, with children in some arms receiving supplements from 6 to 18 or 24 months of age (described in detail in the methods section).

The effects of these interventions on index child and maternal outcomes varied across the two trials. In Bangladesh, maternal supplementation with LNS during pregnancy improved several birth outcomes, including a reduction in the prevalence of newborn stunting (Mridha et al., 2016), and comprehensive LNS supplementation (maternal supplementation during pregnancy and the first 6 months postpartum plus child supplementation from 6 to 24 months of age) improved child growth and development through 24 months of age (Dewey et al., 2017; Matias et al., 2017). In the mothers, those in the LNS group experienced less household food insecurity (Adams et al., 2017) and improved anthropometric indices during pregnancy among those >25 years (Matias et al., 2016), but no differences in vitamin A status during pregnancy (Dewey et al., 2016) or in anaemia, iron status, or iodine status at 6 months postpartum (Matias et al., 2018; Mridha et al., 2017). In Ghana, maternal supplementation with LNS during pregnancy increased birth size, particularly among infants of first‐time mothers (Adu‐Afarwuah et al., 2015), and mean attained length at 18 months of age was higher among children in the comprehensive maternal and child LNS supplementation group (Adu‐Afarwuah et al., 2016). In the mothers, those in the LNS group were less likely to experience inadequate weight gain during pregnancy (Adu‐Afarwuah et al., 2017a) and had improved essential fatty‐acid status during pregnancy and postpartum (Oaks et al., 2017), and those who received LNS or MMN had improved vitamin B_12_ (Shahab‐Ferdows et al., 2019) and iodine status (Adu‐Afarwuah et al., 2018) but did not differ in anaemia, iron status, or breast milk vitamin A concentration at 6 months postpartum or in household food insecurity, compared with those who received IFA (Adams et al., 2017; Adu‐Afarwuah et al., 2017b; Klevor et al., 2016).

As part of the follow‐up to the randomized trials, we collected anthropometric data on the next child born after the index pregnancy and evaluated the impact of maternal supplementation with LNS or with MMN, compared with maternal supplementation with IFA, on the growth status of these younger siblings. We hypothesized that maternal supplementation with LNS, or with MMN, during pregnancy and the first 6 months postpartum, compared with maternal supplementation with IFA during pregnancy only or during pregnancy and the first 3 months postpartum, may have led to improved maternal nutritional status at the conception of the subsequent child, thereby improving the younger siblings' growth status.

## 2 METHODS

### 2.1 Randomized trials

The RDNS trial was implemented by University of California, Davis, in partnership with the International Center for Diarrheal Diseases Research, Bangladesh, LAMB, and the Food and Nutrition Technical Assistance III Project of Family Health International 360. RDNS was a cluster‐randomized effectiveness trial conducted in 11 rural unions of the Badarganj and Chirirbandar subdistricts in the northwest region of Bangladesh. LAMB, one of the implementing partners, was a nongovernmental organization that provided community‐based health services to women and children in the study area. LAMB community health workers delivered the supplements to women and children who participated in the trial, and study clusters were defined based on the LAMB community health workers' 64 work areas.

Pregnant women who were 20 weeks of gestation or less were enrolled into the RDNS trial on a rolling basis between October 2011 and August 2012. Enrolled women (*n* = 4,011) were assigned to one of four intervention groups (defined in Table 1) based on their residence within one of the 64 clusters, with 16 randomly assigned clusters per arm. The original RDNS minimum sample size calculation of 778 women per group (total of 3,152 women) was based on detecting an effect size of 0.2 for comparison of continuous outcomes between any two groups with a one‐sided hypothesis test at the 5% level of significance with 80% power and assuming an intracluster correlation of 0.01 (Mridha et al., 2016).

**Table 1:** Rang‐Din Nutrition Study and DYAD‐G intervention groups as defined during the main trials and for follow‐up sibling analyses

RDNS intervention groups	
Main trial	Follow‐up younger sibling analysis^a^
(1) Comprehensive LNS: Women received 20 g of LNS per day through pregnancy and the first 6 months postpartum, and their children received 20 g of LNS per day from 6–24 months of age.	(1) LNS (Comprehensive LNS)
(2) Child‐only LNS: Women received daily IFA capsules during pregnancy and the first 3 months postpartum, and their children received 20 g LNS from 6–24 months.	(2) IFA (Child‐only LNS + Child‐only MNP + Control)
(3) Child‐only MNP: Women received daily IFA capsules during pregnancy and the first three months postpartum, and their children received micronutrient powder from 6–24 months.	
(4) Control: Women received daily IFA capsules during pregnancy and the first 3 months postpartum (no child supplementation).	
DYAD‐G intervention groups	
Main trial	Follow‐up younger sibling analysis^b^
(1) LNS: Women received 20 g/day LNS during pregnancy and the first 6 months postpartum followed by 20 g/day LNS for the infant from 6 to 18 months of age.	(1) LNS
(2) MMN: Women received daily multiple micronutrient capsules during pregnancy and the first 6 months postpartum and no infant supplementation.	(2) MMN
(3) IFA: Women received daily iron–folic acid capsules during pregnancy, a low‐dose calcium placebo capsule for the first 6 months postpartum and no infant supplementation.	(3) IFA

Abbreviations: IFA, iron–folic acid; LNS, lipid‐based nutrient supplements; MMN, multiple micronutrient; MNP, micronutrient powder.

a The current analysis was conducted using follow‐up data collected several years after the “main trial,” a randomized effectiveness trial in Bangladesh. Intervention groups for the follow‐up analysis were as defined in the “follow‐up younger sibling analysis” column.

b The current analysis was conducted using follow‐up data collected several years after the “main trial,” a randomized efficacy trial in Ghana. Intervention groups for the follow‐up analysis were as defined in the “follow‐up younger sibling analysis” column.

The DYAD‐G trial was implemented by the Lipid‐based Nutrient Supplements Project between December 2009 and March 2014 in the semiurban Yilo Krobo and Lower Manya Krobo districts of the Eastern Region of Ghana. Between December 2009 and December 2011, pregnant women who were 20 weeks of gestation or less and at least 18 years of age were enrolled on a rolling basis. Women were randomly assigned to one of three intervention groups (Table 1). The DYAD‐G sample size calculations were based on detecting an effect size of 0.3 for comparison of continuous outcomes between any two groups with a two‐sided hypothesis test at the 5% level of significance and with 80% power (Adu‐Afarwuah et al., 2015). Because of a temporary mislabelling of the IFA and MMN supplements (described later), additional women were enrolled to allow for sufficient power to assess pregnancy outcomes if women who temporarily received a mislabelled supplement were excluded. In total, 1,320 women were enrolled and randomized (*n* = 440 for the LNS group, *n* = 439 for the MMN group, and *n* = 441 for IFA group; Adu‐Afarwuah et al., 2015).

The nutrient composition of each of the maternal supplements is provided in Table S1 in the online supporting material (OSM). Both main trials received ethical approval and were registered at http://clinicaltrials.gov.

### 2.2 Follow‐up data collection

Several years after the completion of each of the main trials, we carried out follow‐up studies to collect additional data from the mothers who had participated in the trials, the index children, and other members of the index children's families. The RDNS follow‐up occurred from January to August 2016, when the index children were ~40–52 months of age, whereas the follow‐up to the DYAD‐G trial occurred between January and December of 2016 when the index children were 4–6 years of age. All of the women who participated in the main trials as well as the index children born during the main trials were eligible to participate in the follow‐up study.

As a component of the follow‐up data collection activities in both countries, we collected anthropometric data on the closest younger living sibling born after the index child. All closest younger siblings born after the index children were eligible for follow‐up anthropometric measurements with several exceptions. We did not attempt to collect sibling anthropometric data in cases where the woman who participated in the main trial or the index child had died, nor did we attempt follow‐up data collection when the woman had a misdiagnosed pregnancy, miscarriage, or stillbirth during the main trial. In cases of younger sibling twins, both twins were measured, and one twin was randomly selected for inclusion in the younger sibling analyses.

For eligible younger siblings who participated in the follow‐up study, anthropometric measurements were taken at a central location or in participants' homes. A team of anthropometrists trained to World Health Organization (WHO) standards performed all sibling anthropometric measurements according to WHO standard procedures (de Onis et al., 2004). The recumbent length of siblings under 24 months of age (or under 85 cm in DYAD‐G) was measured to the nearest 0.1 cm using an infantometer, whereas standing height of those 24 months of age and older was measured to the nearest 0.1 cm using a stadiometer. For all younger siblings, weight was measured to the nearest 50 g using an electronic scale (Seca 874), and a Shorr tape was used to measure mid‐upper arm circumference and head circumference to the nearest 0.1 cm.

In Ghana, anthropometric data were entered into tablets that were preprogrammed using Open Data Kit software. The Open Data Kit data entry forms included preprogrammed quality checks to ensure data were entered for all relevant questions/measurements and to prevent entry of implausible values. All measurements were performed twice and, once entered into the form, were automatically compared for agreement. If the difference between the first two measurements exceeded a predefined threshold (>0.5 cm for length/height, mid‐upper arm circumference, and head circumference; >0.1 kg for weight), the anthropometrists received a software‐generated prompt to take and enter a third measurement. In Bangladesh, anthropometric data were collected on paper forms. The difference in the first two anthropometric measurements was manually calculated, and a third measurement was taken if the difference between the first two measurements exceeded the same predefined thresholds as were used in Ghana.

The University of California, Davis and the Center for Diarrheal Diseases Research, Bangladesh provided ethical approval for the RDNS follow‐up study as a modification to the main study protocol. The DYAD‐G follow‐up study received approval from the ethics committees of the University of California, Davis, the Ghana Health Service, and the University of Ghana College of Basic and Applied Sciences. Prior to participation in both follow‐up studies, mothers (or caregivers) provided written informed consent.

### 2.3 Younger sibling subsample and statistical analysis

Our analyses assessed the impact of maternal LNS or MMN supplementation during the index pregnancy and for the first 6 months postpartum on the growth of the next living child conceived after the index pregnancy. To evaluate this impact, we generated younger sibling anthropometric outcomes on the basis of the WHO 2006 Growth Standards (WHO, 2006). These outcomes were length‐for‐age *z* score/height‐for age *z* score, weight‐for‐age *z* score, weight‐for‐length *z* score/weight‐for‐height *z* score, body mass index (BMI)‐for age *z* score, head circumference‐for‐age *z* score (HCZ), and mid‐upper arm circumference‐for‐age *z* score. We defined sibling stunting as length‐for‐age *z* score/height‐for age *z* score < −2, wasting as weight‐for‐length *z* score/weight‐for‐height *z* score < −2, and underweight as weight‐for‐age *z* score< −2.

We developed a statistical analysis plan prior to conducting the analyses. All analyses were performed using SAS version 9.4 (SAS Inst. Cary, NC, USA). Hypothesis tests were two‐sided at the 5% level of significance. On the basis of the full samples of women initially randomized into the main trials, we began by assessing the overall and group‐specific rates of attrition at follow‐up. We tested for by‐group differences in attrition and summarized and compared the background characteristics between the follow‐up and lost to follow‐up samples. We also assessed by‐group differences in the proportion of index children who had a younger sibling in the follow‐up sample.

We assessed balance in three ways. First, we summarized and compared the baseline characteristics of the follow‐up and lost to follow‐up samples, which were defined based on the initial randomizations. Next, among the full follow‐up sample, we summarized and tested for differences in background characteristics between those with and without a younger sibling. Finally, among the follow‐up sample with a younger sibling, we summarized background characteristics and tested for differences in these characteristics by intervention group. Background characteristics included a household asset index score and the household food insecurity access scale score. Principal components analysis was used to construct the asset index based on household ownership of a set of assets, housing characteristics, and water and sanitation sources, where a higher score indicated a relatively better socioeconomic status (Vyas & Kumaranayake, 2006). The household food insecurity access scale score, which ranged from 0 to 27 with a higher score indicating more severe food insecurity, was based on self‐reported answers about the frequency of experiencing each of nine food insecurity access conditions (Coates, Swindale, & Bilinsky, 2007).

The primary analyses were by intention‐to‐treat, that is, by‐group analyses were according to group assignment regardless of any protocol violations. We also performed a secondary per‐protocol analysis on the basis of self‐reported maternal adherence to her assigned supplement during the main trial. In particular, the per‐protocol samples were limited to the next youngest children of women with at least 60% reported adherence during pregnancy and the period of postpartum supplementation.

For RDNS, the by‐group primary and per‐protocol analyses compared two groups on the basis of assigned maternal supplement (Table 1): LNS (comprehensive LNS groups) versus IFA (combined child‐only LNS, child‐only micronutrient powder, and control groups). Exploratory analyses were also conducted to test for differences among the four child intervention arms. The DYAD‐G primary and per‐protocol analyses were based on tests for differences among the three intervention groups (LNS, MMN, and IFA). When the global null hypothesis of no difference between the three groups was rejected at the 5% level, we performed post hoc pairwise group comparisons corrected for multiple hypothesis testing using the Tukey–Kramer adjustment. We also conducted two additional sets of DYAD‐G analyses. First, where no differences between the IFA and MMN groups were found, we conducted secondary analyses to test for differences between the LNS group and the combined IFA and MMN groups. Second, during the main trial, a temporary mislabelling of the capsules provided to women during pregnancy (described in Adu‐Afarwuah et al., 2015) meant that 170 women assigned to the IFA group actually received MMN capsules for part or all of their pregnancy (and IFA for the remainder of the maternal supplementation period), whereas 170 women assigned to the MMN group actually received IFA capsules for part or all of their pregnancy (then MMN for the remainder of the maternal supplementation period). We therefore conducted an additional “as received” analyses in which group assignments corresponded to the supplements women actually received at enrollment.

The statistical models estimated to detect by‐group differences in sibling anthropometric outcomes were site‐specific to account for the differing designs of each of the main trials. For the RDNS follow‐up sibling sample, continuous outcomes were modelled using mixed‐model linear regression, whereas mixed‐model logistic regressions were used to analyse dichotomous sibling outcomes. Each of the mixed models accounted for the regionally cluster‐randomized design of the main RDNS trial by including the effect of union (nested within subdistrict) and the random effect of cluster (nested within intervention group). With our RDNS observed sample size of 472 younger siblings and assuming a two‐sided hypothesis test at the 5% level of significance with 80% power and an intracluster correlation of 0.01, in two‐group comparisons, we had sufficient power to detect a mean difference of >0.36 *SD* for continuous outcomes, and using the control group stunting rate as the reference prevalence of stunting, a difference of >16% in stunting.

Randomization in the DYAD‐G main trial was at the individual level, so continuous and dichotomous outcomes for younger siblings in the DYAD‐G follow‐up sample were modelled using ordinary least squares and logistic regression, respectively. With our DYAD‐G observed sample size of roughly 300 younger siblings and again assuming a two‐sided hypothesis test at the 5% level of significance with 80% power, we had sufficient power to detect a mean difference of >0.36 *SD* in two group comparisons and a mean difference of >0.46 *SD* in three‐group comparisons of continuous outcomes. Using the control group stunting rate as the reference prevalence of stunting, we had power to detect a difference in stunting of >12% in two‐group comparisons and >14% in three‐group comparisons.

For both RDNS and DYAD‐G, all models included a fixed‐effect control for sibling age at the time of measurement. Fully adjusted analyses included sibling age at the time of measurement in addition to other prespecified covariates, potentially including maternal age at enrollment, maternal years of education at enrollment, maternal height at enrollment, predicted maternal prepregnancy BMI, maternal parity at enrollment, household asset index score, household food insecurity access scale score, younger sibling age at growth measurement, younger sibling sex, and birth interval between index child and younger sibling. Among the potential covariates, only those that were at least marginally associated (*p* < .1) with a specific sibling anthropometric outcome in bivariate analysis were included in the fully adjusted regression model for that outcome.

## 3 RESULTS

### 3.1 Attrition and balance

The RDNS main trial enrolled 4,011 women. Data of any kind were collected from 3,440 households at the 40–52‐month follow‐up, resulting in an overall attrition rate of 14.2%, which did not differ between intervention groups (14.1% in the LNS group vs. 14.3% in the IFA group, *p* = .940). Excluding households that were not targeted for follow‐up because of miscarriage or stillbirth during the main trial or because the index child had died before the end of the main trial, the rate of successful follow‐up was 98.3% (3,440/3,499).

The DYAD‐G main trial enrolled 1,320 women, and follow‐up data of any kind were collected from 1,040 households at the 4–6‐year follow‐up. The overall DYAD‐G attrition rate at follow‐up was therefore 21.2%, which was marginally significantly different between intervention groups (17.7% in the LNS group vs. 22.8% in the MMN group vs. 23.1% in the IFA group, *p* = .092). Again excluding households that were not targeted for follow‐up (due to miscarriage, stillbirth, or index‐child death during the main trial), the DYAD‐G rate of successful follow‐up was 85.1% (1,040/1,222).

The baseline characteristics of the follow‐up and lost to follow‐up samples are summarized and compared in Table S2. In both the RDNS (top panel) and DYAD‐G (bottom panel) samples, there were statistically significant differences in the characteristics of the follow‐up and lost to follow‐up samples, though in all cases, the magnitude of the differences was small.

Of the 3,440 households in the RDNS follow‐up sample, 472 (13.7%) had a younger sibling with anthropometric data. Among households in the overall follow‐up sample, 16.1% in the LNS group had a younger sibling compared with 12.9% in the IFA group (*p* = .04). In Ghana, 327 of the 1,440 households (31.4%) in the DYAD‐G follow‐up sample had a younger sibling with anthropometric data, which did not differ by group (29.8% [LNS] vs. 34.2% [MMN] vs. 30.4% [IFA], *p* = .569). Table 2 summarizes and compares the background characteristics of these two groups (those with a younger sibling with anthropometric data and those without a younger sibling) within the follow‐up samples. In the RDNS follow‐up sample, compared with those without a younger sibling in the follow‐up sample, the mothers of those with a younger sibling were younger and more likely to be nulliparous when they enrolled in the main trial and had a slightly lower prepregnancy (prior to the index pregnancy) BMI and slightly lower household asset index scores. In the DYAD‐G follow‐up sample, households with a younger sibling tended to include mothers who were younger when they enrolled in the main trial, mothers who were slightly taller, and mothers for whom the first child was the index child.

**Table 2:** Baseline characteristics of follow‐up samples with and without a younger sibling

Trial	Variable	Has younger sibling			No younger sibling		*p* value^b^
		*N*	*M* ± *SD* or %^a^		N	*M* ± *SD* or %^a^	
RDNS	Maternal age (y)	472	20.2 ± 3.8		2968	22.2 ± 5.0	<.001
	Maternal education (y)	472	6.0 ± 3.1		2968	6.3 ± 3.3	.091
	Maternal height (cm)	472	150.4 ± 5.4		2968	150.7 ± 5.4	.238
	Maternal prepregnancy BMI (kg/m^2^)	472	19.8 ± 2.6		2968	20.0 ± 2.7	.029
	Mother nulliparous at index pregnancy (%)	472	58.5		2965	36.5	<.001
	Household asset index score	472	−0.2 ± 2.2		2968	0.1 ± 2.3	.008
	HFIAS score (0–27)	472	3.3 ± 4.3		2968	2.9 ± 3.9	.137
DYAD‐G	Maternal age (y)	327	25.7 ± 4.6		713	27.3 ± 5.7	<.001
	Maternal education (y)	327	7.9 ± 3.5		713	7.6 ± 3.6	.220
	Maternal height (cm)	323	159.5 ± 5.6		700	158.7 ± 5.7	.050
	Maternal prepregnancy BMI (kg/m^2^)	322	24.3 ± 4.1		700	24.7 ± 4.6	.111
	Mother nulliparous at index pregnancy (%)	327	37.9		713	29.6	.008
	Household asset index score	327	0.0 ± 1.0		710	0.0 ± 1.0	.678
	HFIAS score (0–27)	325	2.3 ± 3.8		708	2.7 ± 4.4	.116

*Note*. The values of baseline characteristics are from the baseline of the main randomized trials.

Abbreviations: BMI, body mass index; HFIAS, household food insecurity access scale.

a Values are *M* ± *SD* for continuous variables and percentage for dichotomous variables.

b For continuous variables, *p* values for tests of difference in mean between follow‐up samples with and without a younger sibling based on mixed model linear regressions (RDNS) or ordinary least squares regressions (DYAD‐G). For dichotomous variables, *p* values for tests of difference in proportion between follow‐up samples with and without a younger sibling based on mixed model logistic regressions (RDNS) or logit regressions (DYAD‐G).

Table 3 shows that the intervention groups were balanced in terms of baseline characteristics and background characteristics (sex and birth interval) of the younger siblings in both the RDNS and DYAD‐G follow‐up samples of younger siblings with anthropometric measurements. The average birth interval between the index child and the younger sibling was ~30 months in the RDNS sample and ~39 months in the DYAD‐G sample.

**Table 3:** Characteristics of follow‐up sample with younger sibling by intervention group

Trial	Variable	LNS			IFA			MMN		*p* value^c^
		*N*	*M* ± *SD* or %^b^		*N*	*M* ± *SD* or %^b^		*N*	*M* ± *SD* or %^b^	
RDNS	Maternal age^a^ (y)	145	19.9 ± 3.4		327	20.3 ± 4.0				.301
	Maternal education^a^ (y)	145	5.9 ± 2.5		327	6.1 ± 3.3				.777
	Maternal height^a^ (cm)	145	150.7 ± 5.5		327	150.3 ± 5.4				.443
	Maternal prepregnancy BMI^a^ (kg/m^2^)	145	19.5 ± 2.5		327	19.9 ± 2.6				.185
	Mother nulliparous^a^ (%)	145	57.2		327	49.6				.718
	Household asset index score^a^	145	−0.4 ± 1.8		327	‐0.1 ± 2.3				.159
	HFIAS score^a^ (0–27)	145	3.2 ± 4.2		327	3.3 ± 4.4				.993
	Sibling female (%)	145	52.4		327	52.0				.929
	Birth interval between index child and sibling (m)	145	30.1 ± 7.9		327	29.7 ± 8.3				.644
	Sibling age at measurement (m)	145	13.6 ± 8.1		326	13.8 ± 8.4				.787
DYAD‐G	Maternal age^a^ (y)	108	25.7 ± 4.5		103	25.4 4.8		116	26.0 ± 4.5	.627
	Maternal education^a^ (y)	108	8.1 ± 3.7		103	8.1 3.7		116	7.4 ± 3.2	.250
	Maternal height^a^ (cm)	106	159.6 ± 5.2		102	159.1 6.4		115	159.7 ± 5.3	.709
	Maternal prepregnancy BMI^a^ (kg/m^2^)	106	24.7 ± 4.1		102	23.7 3.7		114	24.4 ± 4.4	.242
	Mother nulliparous^a^ (%)	108	42.6		103	40.8		116	31.0	.159
	Household asset index score^a^	108	−0.1 ± 1.0		103	0.1 1.1		116	0.0 ± 1.0	.193
	HFIAS score^a^ (0–27)	107	2.2 ± 3.8		103	1.8 3.4		115	2.6 ± 4.0	.285
	Sibling female (%)	99	38.4		99	47.5		109	43.1	.435
	Birth interval between index child and sibling (m)	108	39.9 ± 12.7		103	36.5 ± 11.1		116	38.7 ± 12.0	.116
	Sibling age at measurement (m)	108	22.4 ± 13.0		103	24.3 ± 12.9		116	24.6 ± 12.7	.285

Abbreviations: BMI, body mass index; HFIAS, household food insecurity access scale; IFA, iron–folic acid; LNS, lipid‐based nutrient supplements; MMN, multiple micronutrient; RDNS, Rang‐Din Nutrition Study.

a Denotes baseline characteristics from the original randomized trial.

b Values are *M* ± *SD* for continuous variables and percentage for dichotomous variables.

c For continuous variables, *p* values for tests of difference in mean between groups based on mixed model linear regressions (RDNS) or ordinary least squares regressions (DYAD‐G). For dichotomous variables, *p* values for tests of difference in proportion between groups based on mixed model logistic regressions (RDNS) or logit regressions (DYAD‐G).

### 3.2 Younger sibling growth

The effects of the intervention on continuous and dichotomous sibling growth status outcomes are shown in Tables 4 and 5, respectively. Among younger siblings in the RDNS follow‐up sample, who were on average 13.8 months (standard deviation of 8.3) of age at measurement, there were no significant differences in any of the continuous growth status outcomes between siblings of mothers who received LNS in the previous pregnancy and the first 6 months postpartum compared with siblings of mothers who received IFA. Likewise, there were no differences in the prevalence of stunting, wasting, or underweight between the two groups. Tables S3 and S4 in the OSM show that, comparing the four RDNS child intervention arms, there were no differences in the continuous and dichotomous outcomes, respectively. The observed intracluster correlation for each outcome was essentially zero.

**Table 4:** Continuous younger sibling anthropometric outcomes

Trial	Variable	LNS			IFA			MMN		*p* value^b^
		*N*	Mean (95% CI)		*N*	Mean (95% CI)		*N*	Mean (95% CI)	
RDNS	HAZ/LAZ	144	−1.44 [−1.66, −1.22]		325	−1.54 [−1.70, −1.38]				.398
	WAZ	145	−1.39 [−1.59, −1.19]		326	−1.40 [−1.54, −1.26]				.945
	WHZ/WLZ	144	−0.81 [−0.97, −0.65]		325	−0.74 [−0.84, −0.64]				.494
	BMIZ	144	−0.79 [−0.93, −0.65]		325	−0.66 [−0.76, −0.56]				.160
	HCZ	145	−1.61 [−1.77, −1.45]		326	−1.64 [−1.76, −1.52]				.794
	MUACZ^a^	127	−0.53 [−0.71, −0.35]		293	−0.51 [−0.63, −0.39]				.862
DYAD‐G	HAZ/LAZ	85	−0.87 [−1.11, −0.63]		94	−0.91 [−1.15, −0.67]		96	−0.91 [−1.15, −0.67]	.964
	WAZ	86	−0.57 [−0.79, −0.35]		90	−0.54 [−0.76, −0.32]		94	−0.68 [−0.90, −0.46]	.626
	WHZ/WLZ	85	−0.13 [−0.35, 0.09]		90	−0.04 [−0.26, 0.18]		94	−0.25 [−0.45, −0.05]	.357
	BMIZ	85	−0.03 [−0.25, 0.19]		90	0.07 [−0.15, 0.29]		94	−0.16 [−0.36, 0.04]	.322
	HCZ	98	−0.84 [−1.04, −0.64]		99	−0.55 [−0.75, −0.35]		107	−0.71 [−0.89, −0.53]	.102
	MUACZ^a^	81	−0.44 [−0.62, −0.26]		86	−0.38 [−0.56, −0.20]		95	−0.58 [−0.74, −0.42]	.211

Abbreviations: BMIZ, body mass index‐for age *z* score; HAZ/LAZ, height‐for‐age *z* score/length‐for‐age *z* score; HCZ, head circumference‐for‐age *z* score; IFA, iron–folic acid; LNS, lipid‐based nutrient supplements; MMN, multiple micronutrient; MUACZ, mid‐upper arm circumference‐for‐age *z* score; WAZ, weight‐for‐age *z* score; WHZ/WLZ, weight‐for‐height *z* score/weight‐for‐length *z* score.

a MUACZ calculated for children 3 months of age and older.

b All models control for younger sibling age at growth measurement.

**Table 5:** Dichotomous younger sibling anthropometric outcomes

Trial	Variable	LNS			IFA			MMN		*p* value^a^
		*N*	Prevalence (95% CI)		*N*	Prevalence (95% CI)		*N*	Prevalence (95% CI)	
RDNS	Stunted	144	29.2 [23.9, 34.4]		325	34.2 [28.3, 40.0]				.439
	Wasted	144	7.6 [3.8, 11.5]		325	9.2 [6.7, 11.8]				.603
	Underweight	145	24.1 [18.3, 30.0]		326	28.5 [22.7, 34.3]				.418
DYAD‐G	Stunted	85	9.4 [3.2, 15.6]		94	20.2 [12.1, 28.3]		96	13.5 [6.68, 20.4]	.139
	Wasted	85	1.2 [−1.1, 3.5]		90	3.3 [−0.4, 7.1]		94	2.1 [‐0.79, 5.1]	.600
	Underweight	86	5.8 [0.9, 10.8]		90	12.2 [5.4, 19.0]		94	11.7 [5.19, 18.2]	.305

*Note*. Stunted defined as height‐for‐age *z* score/length‐for‐age *z* score < −2. Wasted defined as weight‐for‐height *z* score/weight‐for‐length *z* score < −2. Underweight defined as WAZ < −2.

Abbreviations: IFA, iron–folic acid; LNS, lipid‐based nutrient supplements; MMN, multiple micronutrient; RDNS, Rang‐Din Nutrition Study.

a All models control for younger sibling age at growth measurement.

Among younger siblings in the DYAD‐G follow‐up sample, who were on average 21.2 months (standard deviation of 13.4) of age at measurement, the continuous and dichotomous growth status outcomes were not significantly different among the LNS, MMN, and IFA groups. Notably, however, there was a substantial difference in the prevalence of stunting in the LNS group compared with the IFA group (9.4% in the LNS group vs. 20.2% in the IFA group; relative risk 0.53 [0.25, 1.46]), though the difference was not statistically significant (*p* = .139). Tables S5 and S6 show the DYAD‐G results comparing the LNS group with the combined IFA and MMN groups. There were no differences between these two groups in any of the continuous or dichotomous outcomes, but again, there was a notable difference that did not reach statistical significance in stunting between groups (9.4% in the LNS group vs. 16.8% in the combined IFA + MMN group, *p* = 095).

As shown in Tables S7 and S8 in the OSM, these findings did not change for either RDNS or DYAD‐G based on the fully adjusted models that controlled for predefined baseline covariates. The results of the per‐protocol analyses are shown in Tables S9 and S10 in the OSM. Among just the sample of siblings for whom the mother reported consuming her assigned supplement on at least 60% of the days during pregnancy and the postpartum period of supplementation, there were no group differences in any of the continuous or dichotomous outcomes in either the RDNS or DYAD‐G samples.

Finally, Tables S11 and S12 in the OSM show the effects on the continuous and dichotomous, respectively, DYAD‐G sibling growth status outcomes on the basis of the “as received” analyses in which group assignments correspond to the supplements women actually received at enrollment in Ghana. These results showed a difference in the mean HCZ among groups (*p* = .042). Pairwise group comparisons, corrected for multiple hypothesis testing using the Tukey–Kramer adjustment, showed the mean HCZ was significantly lower in the LNS group compared with the MMN group (estimated difference in mean HCZ of −0.32 [−0.63, −0.01], *p* = .043).

## 4 DISCUSSION

Although there is increasing recognition of the potential importance of maternal preconception nutrition for birth outcomes and child growth, there is relatively little evidence directly evaluating the effect of interventions that improve maternal nutrition during the preconception period on these outcomes. The analyses presented here evaluated the potential for longer term benefits of maternal supplementation during pregnancy and postpartum on the growth status of the next child born after the index pregnancy/postpartum period. In both Bangladesh and Ghana, overall we found no differences in growth status or the prevalence of undernutrition among younger siblings whose mothers had received LNS (or MMN in Ghana) during the index pregnancies and postpartum periods, compared with younger siblings of mothers who had received IFA. However, in both Bangladesh and Ghana, the prevalence of stunting was lower (5 percentage points in Bangladesh and >10 percentage points in Ghana) among siblings whose mothers had received LNS compared with those who had received IFA, though we had relatively low power to detect differences in dichotomous outcomes and these differences in the prevalence of stunting were not statistically significant.

Other available evidence, from three trials that started preconception maternal supplementation at least 3 months prior to conception, is mixed. A recent multicountry (Democratic Republic of the Congo, Guatemala, India, and Pakistan) randomized controlled trial showed no difference in birth outcomes in the group that received daily LNS starting at least 3 months before conception compared with the group that received LNS starting late in the first trimester of pregnancy, though birth outcomes in both LNS groups were improved relative to the control group that did not receive supplementation (Hambidge et al., 2019). An earlier food‐based intervention in India showed no difference in birth weight among the infants of women who were provided with a nutrient‐dense daily snack prior to conception and through pregnancy, compared with the infants of women provided a similar snack but with low micronutrient content (control; Potdar et al., 2014). However, among women who began consuming the snack 90 days or more before their last menstrual period, birth weight was higher in the nutritious snack group compared with the control group. Finally, in a study in Vietnam that provided preconception women with either daily IFA, daily MMN, or daily folic acid supplements, there were no differences in birth outcomes in either the group that received MMN or the group that received IFA compared with the group that received folic acid, even among the subgroup of women who received their assigned supplement for at least 26 weeks before conception (Ramakrishnan et al., 2016). However, a follow‐up to that study found that, at 2 years of age, the children of women who received preconception MMN or preconception IFA had improved linear growth and reduced risk of stunting compared with those who received only folic acid (Nguyen et al., 2017).

Our findings of no intervention group differences in growth status or the prevalence of undernutrition among younger siblings have several possible explanations. The first is that the control group in both trials received IFA, which by itself may have had a positive impact on maternal nutritional status prior to conception of the next child. In the trial in Vietnam (Nguyen et al., 2017), preconception MMN and IFA were both shown to improve linear growth at 2 years of age compared with preconception folic acid alone. In our trials, the daily dose of iron contained in the IFA capsules was 60 mg, whereas the daily dose of iron in the MMN capsules and the LNS was 20 mg (Table S1). Thus, it is possible that there was no detectable advantage of LNS or MMN beyond the effect of the IFA. Another possibility is that improved maternal nutritional status during the trials was not sustained through conception with her next child. It is possible, for example, that after 6 months postpartum and the end of maternal supplementation, continued demands on maternal nutrient stores during the remainder of lactation could have depleted stores of some nutrients, such as fat‐soluble vitamins. On average, the gap between the end of maternal supplementation at 6 months postpartum and conception of her next child was ~15 months in the RDNS sample and ~25 months in the DYAD‐G sample. Therefore, any effect of improved maternal nutritional status at 6 months postpartum may have been diminished by the time the younger sibling was conceived.

This analysis had several strengths. First, the rate of successful follow‐up was high for both studies (~98% in RDNS and ~85% in DYAD‐G). Another strength was that the differences between the study sites in Bangladesh and Ghana improved the external validity of our results. Bangladesh and Ghana have differing background rates of maternal and child undernutrition: In Bangladesh in 2014, 36% of children under 5 years of age were stunted, and 31% of ever‐married women of reproductive age had low BMI (BMI < 18.5; National Institute of Population Research and Training (NIPORT), Mitra and Associates, & ICF International, 2016), whereas in Ghana in 2014, the prevalence of stunting among children under age 5 was 19%, and prevalence of low BMI among women of reproductive age was 6% (Ghana Statistical Service, Ghana Health Service, & ICF International, 2015). The external validity of our results was also strengthened due to the study samples being drawn from different populations in each country (rural in Bangladesh and semiurban in Ghana) as well as the differing study designs between the two countries, with the RDNS study designed as an effectiveness trial and DYAD‐G as an efficacy trial.

The results of these analyses are also qualified by several limitations. First, it is possible that some women in our samples had one or more pregnancy between the index child and the younger sibling included in our analyses that ended in miscarriage, stillbirth, or child death. Pregnancy histories were collected at follow‐up for the RDNS sample, and among those with a child included in this analysis, 4% reported more than one pregnancy after the index child. Although similar data are not available for DYAD‐G, the rate is likely a bit higher given that more time elapsed between the end of maternal supplementation during the main trial and follow‐up data collection. In these cases, the pathway between the nutritional status of the mother during the index pregnancy and postpartum period and the growth status of her next living child is less direct. Another limitation is that having another child after the index child was a choice variable, resulting in a selected (and relatively small) sample of siblings at follow‐up. In the RDNS sample, there was a significant by‐group difference in the likelihood of having a younger sibling who was measured at follow‐up (no difference in the DYAD‐G sample), and in both samples, there were differences in the background characteristics of those with and without a younger sibling measured at follow‐up. Some of these differences, which included maternal age, maternal parity, maternal BMI, and house asset index score in the RDNS sample and maternal parity, maternal age, and maternal height in the DYAD‐G sample, were expected (i.e., we would expect older mothers and multiparous mothers to be less likely to have another child). The other differences were generally small in magnitude, and these differences, along with other background characteristics, were well balanced among intervention groups in the follow‐up samples. There were also differences in the characteristics of the follow‐up and lost to follow‐up samples, though the magnitude of the differences were small. These differences may have implications with regard to the generalizability of the findings. However, because the follow‐up sample of younger siblings was balanced (i.e., no differences in background characteristics between intervention groups), we consider the results to be internally valid. Finally, it is possible that improved maternal nutrition during a previous pregnancy and postpartum period might have improved a mother's nutritional status during a subsequent pregnancy, resulting in improved birth outcomes for the subsequent child. Because we do not have any information on maternal nutrition during her pregnancy with the younger sibling, nor on younger siblings' birth outcomes, we were unable to assess this possibility.

For the past decade, the global nutrition community has been focused on assessing and implementing nutrition interventions during the first 1,000 days in an effort to prevent the detrimental short‐ and long‐term impacts of undernutrition during this critical window. Increasingly, however, attention is broadening beyond the first 1,000 days to include the preconception period and the potential for interventions that improve preconception maternal nutrition to translate into improved child outcomes beyond what is achievable with interventions that start after conception. This study was unique in that it evaluated the potential for a nutrition intervention during the previous pregnancy and postpartum period to impact the growth status of a subsequent child, and it is important to specify what we can and cannot conclude from the results of this evaluation. What we can conclude is that, overall in these specific settings and without additional intervention, the benefits of maternal supplementation with LNS or MMN during pregnancy and the first 6 months postpartum did not extend to the next child in terms of improved growth status beyond any effects of IFA alone. We cannot, however, conclude from these results that improved maternal preconception nutrition does not improve child growth. More research is needed to understand how preconception nutrition interventions can be most effectively designed and targeted. In our studies, women in the control groups received IFA, so we were not able to assess the impact of IFA received preconceptionally on the growth status of the subsequent child. Given the global prevalence of iron‐deficiency anaemia among women of reproductive age, the research agenda should include studies designed to test whether the provision of IFA alone to women of reproductive age, and especially to young women preconceptionally, improves birth and child growth outcomes.

## CONFLICTS OF INTEREST

The authors declare they have no conflicts of interest.

## CONTRIBUTIONS

KGD, SAA, and MKM designed and supervised the parent trials. KGD, SAA, MKM, BMO, and SLM supervised the follow‐up studies. SMK, HO, and MEO coordinated data collection. CDA conducted the data analysis. KPA and KGD interpreted the data. KPA drafted the manuscript, and all authors critically commented on drafts and approved the final manuscript.

## Supporting information

Table S1. Nutrient composition of RDNS and DYAD‐G supplements
Table S2. Baseline characteristics1 of follow‐up and lost to follow‐up samples
Table S3. RDNS continuous younger sibling anthropometric outcomes, four groups
Table S4. RDNS dichotomous younger sibling anthropometric outcomes, four groups
Table S5. DYAD‐G continuous younger sibling anthropometric outcomes, two groups
Table S6. DYAD‐G Dichotomous younger sibling anthropometric outcomes, two groups^1^
Table S7. Adjusted1 continuous younger sibling anthropometric outcomes
Table S8. Adjusted1 dichotomous younger sibling anthropometric outcomes
Table S9. Continuous younger sibling anthropometric outcomes among mothers with high adherence to protocol^1^
Table S10. Dichotomous younger sibling anthropometric outcomes among mothers with high adherence to protocol^1^
Table S11. DYAD‐G continuous younger sibling anthropometric outcomes by maternal supplement received during pregnancy^1^
Table S12. DYAD‐G dichotomous younger sibling anthropometric outcomes by maternal supplement received during pregnancy^1^
