# Supplementary material for: The impact of maternal supplementation during pregnancy and the first 6 months postpartum on the growth status of the next child born after the intervention period: Follow‐up results from Bangladesh and Ghana
Source: Matern Child Nutr. 2020 Feb 5;16(2):e12927. doi: 10.1111/mcn.12927 (PMC7083484; doi:10.1111/mcn.12927)
Supplement: Supplementary file 1 — Table S1. Nutrient composition of RDNS and DYAD‐G supplements Table S2. Baseline characteristics1 of follow‐up and lost to follow‐up samples Table S3. RDNS continuous younger sibling anthropometric outcomes, four groups Table S4. RDNS dichotomous younger sibling anthropometric outcomes, four groups Table S5. DYAD‐G continuous younger sibling anthropometric outcomes, two groups Table S6. DYAD‐G Dichotomous younger sibling anthropometric outcomes, two groups1 Table S7. Adjusted1 continuous younger sibling anthropometric outcomes Table S8. Adjusted1 dichotomous younger sibling anthropometric outcomes Table S9. Continuous younger sibling anthropometric outcomes among mothers with high adherence to protocol1 Table S10. Dichotomous younger sibling anthropometric outcomes among mothers with high adherence to protocol1 Table S11. DYAD‐G continuous younger sibling anthropometric outcomes by maternal supplement received during pregnancy1 Table S12. DYAD‐G dichotomous younger sibling anthropometric outcomes by maternal supplement received during pregnancy1 [file MCN-16-e12927-s001.pdf]

Online Supporting Material to:

The impact of maternal supplementation during pregnancy and the first six months postpartum on the growth status of the next child conceived and born after the intervention period: follow-up results of two randomized controlled trials in Bangladesh and Ghana

Supporting Tables

Supplemental Table 1. Nutrient composition of RDNS and DYAD-G supplements

Nutrient	Chemical form	Maternal LNS	MMN	IFA
Daily Ration, g/day		20		
Total energy, kcal		118		
Protein, g		2.6		
Fat, g		10		
Linoleic acid, g		4.59		
α-Linoleic acid, g		0.59		
Vitamin A, µg RE	Retinyl acetate	800	800	
Vitamin C, mg	L-ascorbic acid	100	100	
Thiamin, mg	Thiamin hydrochloride	2.8	2.8	
Riboflavin, mg	Riboflavin	2.8	2.8	
Niacin, mg	Niacinamide	36	36	
Folic Acid, mg	Pteroyl monoglutamic acid	400	400	400
Pantothenate, mg	Calcium pantothenate	7	7	
Vitamin B-6, mg	Pyridoxine hydrochloride	3.8	3.8	
Vitamin B-12, µg	Cyanocobalamin (0.1%)	5.2	5.2	
Vitamin D, IU	Cholecalciferol (D3)	400	400	
Vitamin E, mg	DL-alpha-tocopherol acetate	20	20	
Vitamin K, µg	Phylloquinone 5%	45	45	
Iron, mg	Encapsulated ferrous sulphate	20	20	60
Zinc, mg	Zinc sulphate	30	30	
Cu, mg	Encapsulated copper sulphate	4	4	
Calcium, mg	Tricalcium phosphate	280		
Phosphorus, mg	Dipotassium phosphate	190		
Potassium, mg	Potassium chloride	200		
Magnesium, mg	Magnesium citrate	65		
Selenium, µg	Sodium selenite 1.5%	130	130	
Iodine, µg	Potassium iodate	250	250	
Manganese, mg	Manganese sulphate	2.6	2.6	

IFA, iron-folic acid; LNS, lipid-based nutrient supplement; MMN, multiple micronutrient; RE, retinol equivalents.

Supplemental Table 2. Baseline characteristics[1] of follow-up and lost to follow-up samples

Trial	Variable	Follow-up Sample		Lost to Follow-Up Sample		
		N	Mean ± SD or %[2]	N	Mean ± SD or %[2]	P-value[3]
RDNS	Maternal age (y)	3440	21.9 ± 4.9	571	22.3 ± 5.2	0.009
	Maternal education (y)	3440	6.3 ± 3.2	571	5.8 ± 3.3	0.005
	Maternal height (cm)	3440	150.7 ± 5.4	571	149.9 ± 5.3	0.001
	Maternal pre-pregnancy BMI (kg/m^2)	3440	20.0 ± 2.6	571	20.0 ± 2.7	0.453
	Mother nulliparous at index pregnancy (%)	3440	39.5	571	41.4	0.388
	Household asset index score	3440	0.1 ± 2.3	571	-0.3 ± 2.2	0.002
	HFIAS score (0-27)	3440	3.0 ± 4.0	571	3.5 ± 4.2	0.016
DYAD-G	Maternal age (y)	1040	26.8 ± 5.4	280	26.4 ± 5.8	0.214
	Maternal education (y)	1040	7.7 ± 3.6	280	7.5 ± 3.9	0.654
	Maternal height (cm)	1023	159.0 ± 5.7	278	158.2 ± 5.6	0.059
	Maternal pre-pregnancy BMI (kg/m^2)	1022	24.6 ± 4.4	269	24.3 ± 4.2	0.341
	Mother nulliparous at index pregnancy (%)	1040	32.2	280	39.6	0.020
	Household asset index score	1037	0.0 ± 1.0	259	-0.1 ± 1.1	0.277
	HFIAS score (0-27)	1033	2.6 ± 4.2	264	2.9 ± 4.6	0.308

BMI: body mass index; HFIAS: household food insecurity access scale.

[1]The values of baseline characteristics are from the baseline of the main randomized trials.

[2] Values are mean ± standard deviation for continuous variables and percentage for dichotomous variables.

[3] For continuous variables, p-values for tests of difference in mean between follow-up and lost to follow-up sample based on mixed model linear regressions (RDNS) or ordinary least squares regressions (DYAD-G). For dichotomous variables, p-values for tests of difference in proportion between follow-up and lost to follow-up sample based on mixed model logistic regressions (RDNS) or logit regressions (DYAD-G).

Supplemental Table 3. RDNS continuous younger sibling anthropometric outcomes, four groups

	Comprehensive LNS		Child-only LNS		Child-only MNP		Control		
Variable	N	Mean (95% CI)	N	Mean (95% CI)	N	Mean (95% CI)	N	Mean (95% CI)	P-value[1]
HAZ/LAZ	144	-1.44 (-1.66, -1.22)	109	-1.62 (-1.86, -1.38)	121	-1.55 (-1.79, -1.31)	95	-1.43 (-1.68, -1.18)	0.541
WAZ	145	-1.39 (-1.59, -1.19)	109	-1.47 (-1.69, -1.25)	122	-1.39 (-1.61, -1.17)	95	-1.34 (-1.58, -1.10)	0.840
WHZ/WLZ	144	-0.81 (-0.99, -0.63)	109	-0.76 (-0.96, -0.56)	121	-0.74 (-0.92, -0.56)	95	-0.73 (-0.93, -0.53)	0.922
BMIZ	144	-0.78 (-0.94, -0.62)	109	-0.67 (-0.85, -0.49)	121	-0.64 (-0.80, -0.48)	95	-0.66 (-0.84, -0.48)	0.592
HCZ	145	-1.61 (-1.79, -1.43)	109	-1.68 (-1.88, -1.48)	122	-1.63 (-1.81, -1.45)	95	-1.60 (-1.80, -1.40)	0.938
MUACZ[2]	127	-0.53 (-0.71, -0.35)	97	-0.60 (-0.80, -0.40)	111	-0.44 (-0.64, -0.24)	85	-0.50 (-0.72, -0.28)	0.686

HAZ/LAZ, height-for-age z-score/length-for-age z-score; WAZ, weight-for-age z-score; WHZ/WLZ, weight-for-height z-score/weight-for-length z-score; BMIZ, body mass index-for age z-score; HCZ, head circumference-for-age z-score; MUACZ, mid-upper arm circumference-for-age z-score.

[1]MUACZ calculated for children 3 months of age and older.

[2]All models control for younger sibling age at growth measurement.

Supplemental Table 4. RDNS dichotomous younger sibling anthropometric outcomes, four groups

	Comprehensive LNS		Child-only LNS		Child-only MNP		Control		
Variable	N	Mean (95% CI)	N	Mean (95% CI)	N	Mean (95% CI)	N	Mean (95% CI)	P-value[1]
Stunted	144	29.2 (23.9, 34.4)	109	36.7 (28.3, 45.1)	121	34.7 (22.6, 46.9)	95	30.5 (20.5, 40.6)	0.686
Wasted	144	7.6 (3.8, 11.5)	109	10.1 (5.7, 14.5)	121	9.1 (4.8, 13.4)	95	8.4 (4.6, 12.3)	0.923
Underweight	145	24.1 (18.3, 30.0)	109	24.8 (17.0, 32.6)	122	31.2 (18.5, 43.9)	95	29.5 (21.6, 37.5)	0.668

Stunted defined as HAZ/LAZ < -2. Wasted defined as WHZ/WLZ < -2. Underweight defined as WAZ < -2.

[1]All models control for younger sibling age at growth measurement.

Supplemental Table 5. DYAD-G continuous younger sibling anthropometric outcomes, two groups

	LNS		IFA+MMN		
Variable	N	Mean (95% CI)	N	Mean (95% CI)	P-value[2]
HAZ/LAZ	85	-0.87 (-1.11, -0.63)	190	-0.91 (-1.07, -0.75)	0.785
WAZ	86	-0.57 (-0.79, -0.35)	184	-0.61 (-0.77, -0.45)	0.773
WHZ/WLZ	85	-0.13 (-0.35, 0.09)	184	-0.15 (-0.29, -0.01)	0.892
BMIZ	85	-0.03 (-0.25, 0.19)	184	-0.05 (-0.19, 0.09)	0.885
HCZ	98	-0.84 (-1.04, -0.64)	206	-0.63 (-0.77, -0.49)	0.079
MUACZ[1]	81	-0.44 (-0.62, -0.26)	181	-0.49 (-0.61, -0.37)	0.640

HAZ/LAZ, height-for-age z-score/length-for-age z-score; WAZ, weight-for-age z-score; WHZ/WLZ, weight-for-height z-score/weight-for-length z-score; BMIZ, body mass index-for age z-score; HCZ, head circumference-for-age z-score; MUACZ, mid-upper arm circumference-for-age z-score.

[1]MUACZ calculated for children 3 months of age and older.

[2]All models control for younger sibling age at growth measurement.

Supplemental Table 6. DYAD-G Dichotomous younger sibling anthropometric outcomes, two groups[1]

	LNS		IFA+MMN		
Variable	N	Prevalence (95% CI)	N	Prevalence (95% CI)	P-value[2]
Stunted	85	9.4 (3.2, 15.6)	190	16.8 (11.51, 22.17)	0.095
Wasted	85	1.2 (-1.1, 3.5)	184	2.7 (0.37, 5.07)	0.456
Underweight	86	5.8 (0.9, 10.8)	184	12.0 (7.3, 16.7)	0.124

Stunted defined as HAZ/LAZ < -2. Wasted defined as WHZ/WLZ < -2. Underweight defined as WAZ < -2.

[1]All models control for younger sibling age at growth measurement.

Supplemental Table 7. Adjusted[1] continuous younger sibling anthropometric outcomes

		LNS		IFA		MMN		
Trial	Variable	N	Mean (95% CI)	N	Mean (95% CI)	N	Mean (95% CI)	P-value
RDNS	HAZ/LAZ	144	-1.45 (-1.65, -1.25)	325	-1.54 (-1.68, -1.40)			0.409
	WAZ	145	-1.39 (-1.57, -1.21)	326	-1.41 (-1.55, -1.27)			0.856
	WHZ/WLZ	144	-0.79 (-0.95, -0.63)	325	-0.75 (-0.85, -0.65)			0.670
	BMIZ	144	-0.77 (-0.91, -0.63)	325	-0.67 (-0.77, -0.57)			0.262
	HCZ	145	-1.62 (-1.78, -1.46)	326	-1.64 (-1.74, -1.54)			0.761
	MUACZ[2]	127	-0.53 (-0.71, -0.35)	293	-0.52 (-0.64, -0.40)			0.969
DYAD-G	HAZ/LAZ	83	-0.89 (-1.13, -0.65)	93	-0.85 (-1.07, -0.63)	95	-0.93 (-1.15, -0.71)	0.886
	WAZ	84	-0.59 (-0.81, -0.37)	89	-0.46 (-0.68, -0.24)	92	-0.71 (-0.93, -0.49)	0.286
	WHZ/WLZ	83	-0.14 (-0.36, 0.08)	89	0.01 (-0.21, 0.23)	92	-0.26 (-0.46, -0.06)	0.208
	BMIZ	83	-0.04 (-0.26, 0.18)	89	0.11 (-0.11, 0.33)	92	-0.17 (-0.37, 0.03)	0.183
	HCZ	96	-0.80 (-1.00, -0.60)	98	-0.53 (-0.73, -0.33)	105	-0.73 (-0.91, -0.55)	0.128
	MUACZ[2]	79	-0.45 (-0.63, -0.27)	86	-0.36 (-0.52, -0.20)	93	-0.59 (-0.75, -0.43)	0.133

HAZ/LAZ, height-for-age z-score/length-for-age z-score; WAZ, weight-for-age z-score; WHZ/WLZ, weight-for-height z-score/weight-for-length z-score; BMIZ, body mass index-for age z-score; HCZ, head circumference-for-age z-score; MUACZ, mid-upper arm circumference-for-age z-score.

[1]All models potentially nclude controls for maternal age at enrollment, maternal years of education at enrollment, maternal height at enrollment, predicted maternal pre-pregnancy body mass index, maternal parity at enrollment, household asset index, household food insecurity access scale score, younger sibling age at growth measurement, younger sibling sex, and birth interval between index child and younger sibling.

[2]MUACZ calculated for children 3 months of age and older.

Supplemental Table 8. Adjusted[1] dichotomous younger sibling anthropometric outcomes

		LNS		IFA		MMN		
Trial	Variable	N	Prevalence (95% CI)	N	Prevalence (95% CI)	N	Prevalence (95% CI)	P-value
RDNS	Stunted	144	29.2 (23.9, 34.4)	325	34.2 (28.3, 40.0)			0.498
	Wasted	144	7.6 (3.8, 11.5)	325	9.2 (6.7, 11.8)			0.515
	Underweight	145	24.1 (18.3, 30.0)	326	28.5 (22.7, 34.3)			0.412
DYAD-G	Stunted	83	9.6 (3.3, 16.0)	93	20.4 (12.2, 28.6)	95	13.7 (6.8, 20.6)	0.276
	Wasted	85	1.2 (-1.1, 3.5)	90	3.3 (-0.4, 7.1)	94	2.1 (-0.8, 5.1)	0.612
	Underweight	84	6.0 (0.9, 11.0)	89	11.2 (4.7, 17.8)	93	11.8 (5.2, 18.4)	0.352

Stunted defined as HAZ/LAZ < -2. Wasted defined as WHZ/WLZ < -2. Underweight defined as WAZ < -2.

[1]All models potentially include controls for maternal age at enrollment, maternal years of education at enrollment, maternal height at enrollment, predicted maternal pre-pregnancy body mass index, maternal parity at enrollment, household asset index, household food insecurity access scale score, younger sibling age at growth measurement, younger sibling sex, and birth interval between index child and younger sibling.

Supplemental Table 9. Continuous younger sibling anthropometric outcomes among mothers with high adherence to protocol[1]

		LNS		IFA		MMN		
Trial	Variable	N	Mean (95% CI)	N	Mean (95% CI)	N	Mean (95% CI)	P-value[3]
RDNS	HAZ/LAZ	81	-1.47 (-1.74, -1.20)	272	-1.58 (-1.74, -1.42)			0.493
	WAZ	81	-1.41 (-1.66, -1.16)	273	-1.39 (-1.53, -1.25)			0.890
	WHZ/WLZ	81	-0.83 (-1.05, -0.61)	272	-0.71 (-0.83, -0.59)			0.320
	BMIZ	81	-0.80 (-1.00, -0.60)	272	-0.61 (-0.73, -0.49)			0.120
	HCZ	81	-1.62 (-1.84, -1.40)	273	-1.65 (-1.77, -1.53)			0.805
	MUACZ[2]	70	-0.58 (-0.82, -0.34)	245	-0.47 (-0.63, -0.31)			0.370
DYAD-G	HAZ/LAZ	84	-0.87 (-1.12, -0.62)	93	-0.91 (-1.15, -0.67)	96	-0.91 (-1.15, -0.67)	0.965
	WAZ	85	-0.57 (-0.79, -0.35)	89	-0.54 (-0.76, -0.32)	94	-0.68 (-0.90, -0.46)	0.638
	WHZ/WLZ	84	-0.13 (-0.35, 0.09)	89	-0.04 (-0.26, 0.18)	94	-0.25 (-0.47, -0.03)	0.384
	BMIZ	84	-0.03 (-0.25, 0.19)	89	0.06 (-0.16, 0.28)	94	-0.16 (-0.36, 0.04)	0.353
	HCZ	96	-0.85 (-1.05, -0.65)	98	-0.55 (-0.75, -0.35)	107	-0.71 (-0.89, -0.53)	0.084
	MUACZ[2]	80	-0.43 (-0.61, -0.25)	85	-0.39 (-0.57, -0.21)	95	-0.58 (-0.74, -0.42)	0.232

HAZ/LAZ, height-for-age z-score/length-for-age z-score; WAZ, weight-for-age z-score; WHZ/WLZ, weight-for-height z-score/weight-for-length z-score; BMIZ, body mass index-for age z-score; HCZ, head circumference-for-age z-score; MUACZ, mid-upper arm circumference-for-age z-score.

[1]High adherence defined as mothers who reported consuming their assigned supplement on at least 60% of the days during pregnancy and the postpartum period of supplementation.

[2]MUACZ calculated for children 3 months of age and older.

[3]All models control for younger sibling age at growth measurement.

Supplemental Table 10. Dichotomous younger sibling anthropometric outcomes among mothers with high adherence to protocol[1]

		LNS		IFA		MMN		
Trial	Variable	N	Prevalence (95% CI)	N	Prevalence (95% CI)	N	Prevalence (95% CI)	P-value[2]
RDNS	Stunted	81	30.9 (23.1, 38.7)	272	34.56 (27.7, 41.4)			0.900
	Wasted	81	8.6 (3.1, 14.2)	272	9.56 (6.7, 12.5)			0.860
	Underweight	81	28.4 (21.5, 35.3)	273	28.94 (22.6, 35.3)			0.879
DYAD-G	Stunted	84	9.5 (3.2, 15.8)	93	20.43 (12.2, 28.6)	96	13.5 (6.7, 20.4)	0.137
	Wasted	84	1.2 (-1.1, 3.5)	89	3.37 (-0.4, 7.1)	94	2.1 (-0.8, 5.1)	0.600
	Underweight	85	5.9 (0.9, 11.0)	89	12.36 (5.5, 19.2)	94	11.7 (5.2, 18.2)	0.308

Stunted defined as HAZ/LAZ < -2. Wasted defined as WHZ/WLZ < -2. Underweight defined as WAZ < -2.

[1]High adherence defined as mothers who reported consuming their assigned supplement on at least 60% of the days during pregnancy and the postpartum period of supplementation.

[2]All models control for younger sibling age at growth measurement.

Supplemental Table 11. DYAD-G continuous younger sibling anthropometric outcomes by maternal supplement received during pregnancy[1]

	LNS		IFA		MMN		
Variable	N	Mean (95% CI)	N	Mean (95% CI)	N	Mean (95% CI)	P-value[3]
HAZ/LAZ	85	-0.87 (-1.11, -0.63)	90	-1.05 (-1.29, -0.81)	100	-0.79 (-1.01, -0.57)	0.295
WAZ	86	-0.57 (-0.79, -0.35)	87	-0.77 (-0.99, -0.55)	97	-0.47 (-0.69, -0.25)	0.145
WHZ/WLZ	85	-0.13 (-0.35, 0.09)	87	-0.27 (-0.49, -0.05)	97	-0.04 (-0.24, 0.16)	0.307
BMIZ	85	-0.03 (-0.25, 0.19)	87	-0.14 (-0.36, 0.08)	97	0.04 (-0.16, 0.24)	0.468
HCZ	98	-0.84 (-1.04, -0.64)	97	-0.76 (-0.96, -0.56)	109	-0.52 (-0.70, -0.34)	0.042[4]
MUACZ[2]	81	-0.44 (-0.62, -0.26)	81	-0.49 (-0.67, -0.31)	100	-0.48 (-0.64, -0.32)	0.896

HAZ/LAZ, height-for-age z-score/length-for-age z-score; WAZ, weight-for-age z-score; WHZ/WLZ, weight-for-height z-score/weight-for-length z-score; BMIZ, body mass index-for age z-score; HCZ, head circumference-for-age z-score; MUACZ, mid-upper arm circumference-for-age z-score.

[1]During the main trial, a temporary mislabeling of the capsules provided to women during pregnancy meant some women assigned to the IFA group actually received MMN capsules during part or all of their pregnancy, while some women assigned to the MMN group actually received IFA capsules during part or all of their pregnancy. Group assignments for the analyses in this table correspond to the supplements women actually received during the period of mislabeling during pregnancy.

[2]MUACZ calculated for children 3 months of age and older.

[3]All models control for younger sibling age at growth measurement.

[4]LNS vs MMN: -0.32 (0.01, 0.63), p=0.043.

Supplemental Table 12. DYAD-G dichotomous younger sibling anthropometric outcomes by maternal supplement received during pregnancy [1]

	LNS		IFA		MMN		
Variable	N	Prevalence (95% CI)	N	Prevalence (95% CI)	N	Prevalence (95% CI)	P-value[1]
Stunted	85	9.4 (3.2, 15.6)	94	20.2 (12.1, 28.3)	96	13.5 (6.7, 20.4)	0.139
Wasted	85	1.2 (-1.1, 3.5)	90	3.3 (-0.4, 7.1)	94	2.1 (-0.8, 5.1)	0.600
Underweight	86	5.8 (0.9, 10.8)	90	12.2 (5.4, 19.0)	94	11.7 (5.2, 18.2)	0.305

Stunted defined as HAZ/LAZ < -2. Wasted defined as WHZ/WLZ < -2. Underweight defined as WAZ < -2.

[1]During the main trial, a temporary mislabeling of the capsules provided to women during pregnancy meant some women assigned to the IFA group actually received MMN capsules during part or all of their pregnancy, while some women assigned to the MMN group actually received IFA capsules during part or all of their pregnancy. Group assignments for the analyses in this table correspond to the supplements women actually received during the period of mislabeling during pregnancy.

[2]All models control for younger sibling age at growth measurement.
